# Differential Diagnosis of Parotid Gland Tumors: Role of Shear Wave Elastography

**DOI:** 10.1155/2017/9234672

**Published:** 2017-09-13

**Authors:** Jan Heřman, Zuzana Sedláčková, Jaromír Vachutka, Tomáš Fürst, Richard Salzman, Jaroslav Vomáčka, Miroslav Heřman

**Affiliations:** ^1^Department of Otorhinolaryngology, Faculty of Medicine and Dentistry, Palacký University Olomouc and University Hospital Olomouc, IP Pavlova 6, Olomouc, Czech Republic; ^2^Department of Radiology, Faculty of Medicine and Dentistry, Palacký University Olomouc and University Hospital Olomouc, IP Pavlova 6, Olomouc, Czech Republic; ^3^Department of Medical Biophysics, Faculty of Medicine and Dentistry, Palacký University Olomouc, IP Pavlova 6, Olomouc, Czech Republic

## Abstract

**Aim:**

To create a predictive score for the discrimination between benign and malignant parotid tumors using elastographic parameters and to compare its sensitivity and specificity with standard ultrasound.

**Methods:**

A total of 124 patients with parotid gland lesions for whom surgery was planned were examined using conventional ultrasound, Doppler examination, and shear wave elastography. Results of the examinations were compared with those ones of histology.

**Results:**

There were 96 benign and 28 malignant lesions in our cohort. Blurred tumor margin alone proved to be an excellent predictor of malignancy with the sensitivity of 79% and specificity of 97%. Enlarged cervical lymph nodes, tumor vascularisation, microcalcifications presence, homogeneous echogenicity, and bilateral occurrence also discriminated between benign and malignant tumors. However, their inclusion in a predictive model did not improve its performance. Elastographic parameters (the stiffness maxima and minima ratio being the best) also exhibited significant differences between benign and malignant tumors, but again, their inclusion did not significantly improve the predictive power of the blurred margin classifier.

**Conclusion:**

Even though elastography satisfactorily distinguishes benign from malignant lesions on its own, it hardly provides any additional value in evaluation of biological character of parotid gland tumors when used as an adjunct to regular ultrasound examination.

## 1. Introduction

Despite all the available imaging and diagnostic techniques (such as ultrasound, computed tomography, magnetic resonance imaging, and fine needle aspiration cytology (FNAC)), the preoperative diagnosis in salivary gland tumors remains difficult. Some patients with malignant tumors need to undergo a second surgery after the definitive histology is obtained during the first procedure. It could have been avoided, if an accurate diagnosis had been known prior to surgery. In a case of preoperatively suspicious malignancy, the surgeon usually decides for more radical approach.

Ultrasound (US) is the traditional and most frequently used imaging method in patients with salivary gland lesions. Sometimes it is the only imaging method employed before surgery. Several US features of malignant tumors were identified; however, their sensitivity and specificity remain suboptimal [1]. The US-guided FNAC is considered the golden standard in preoperative diagnosis despite its widely recognized limitations.

Elastography is relatively a new way of tissue imaging associated mainly with US. Most studies published lately, exploiting only the older strain elastography, have found that malignant tumors are generally stiffer; that is, the stiffness of malignant tumors is usually higher than that of benign ones. However, the results of elastography in salivary gland lesions have been rather poor so far. A huge overlap between benign and malignant lesions was found in the semiquantitative elastography scores [2–8].

Shear wave elastography (SWE) is a novel elastographic method that offers the advantage of quantitative measurements (tissue stiffness in kPa) and lower operator-dependence and shows a relatively narrow range of normal tissue values [9, 10]. So far, it is well established in breast and thyroid gland lesions [11, 12]. We are aware of three studies only that report the use of SWE in salivary glands [6, 8, 13]. The aim of this study was to calculate the sensitivity and specificity of conventional US and SWE parameters. The secondary aim was to identify a better quantitative elastographic predictor than the traditional semiquantitative elastographic score.

## 2. Materials and Methods

This prospective observational study was approved by the Review Board of Palacký University, Olomouc, under the reference number 153/13 on 16 December 2013.

A total of 124 consecutive patients, for whom parotid tumor surgery was planned, at the ENT Department of the Olomouc University Hospital from January 2014 to February 2017 were referred for ultrasound examination one day prior to the surgery. The cohort comprised 58 women and 66 men aged 15–85 years, with median age of 60 years.

All the patients were examined in supine position by one and only experienced head and neck radiologist (having routinely used the US elastography for more than 5 years) using the Aixplorer US system (SuperSonic Imagine, Aix-en-Provence, France) with a 4–15 MHz compact linear array transducer. The examination consisted of conventional US, Doppler US, and SWE with quantitative assessment (Super Sonic Imaging, tissue stiffness measured in kilopascals). The recorded conventional US features of lesions were as follows: size in three mutually perpendicular dimensions, margin quality (clearly delineated or blurred), shape (lobular or not), homogeneous echogenicity (yes/no), presence of microcalcifications (yes/no) and cystic areas (yes/no), bilaterality (bilateral/unilateral), distal acoustic enhancement (yes/no), acoustic shadow (yes/no), and enlarged neck lymph nodes (yes/no). The number of supplying vessels in the tumor was also assessed using Doppler US and the finding was classified as absent, only peripheral vascularisation, 1-2 vessels, or 3+ vessels.

**Figure 1:**
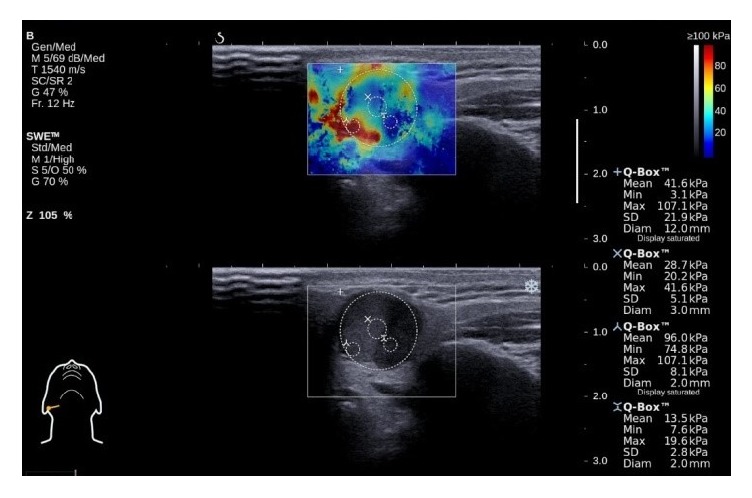
Shear wave elastography assessment with four regions of interest (ROI) marked by circles inside the lesion (parotid pleomorphic adenoma).

The US device with SWE module returns the mean, minimum, maximum, and standard deviation (SD) of the stiffness of a selected region of interest (ROI). For SWE assessment, four ROI were identified. The first circular ROI was drawn with the largest possible diameter not extending beyond the tumor margins. The preset circle size was used for remaining ROI. The second ROI was placed in the very center of the tumor, the third one in the area with the highest stiffness, and the fourth in the area with the lowest stiffness (Figure 1). The minimum value from the lowest stiffness ROI and the maximum from the stiffest ROI did not differ from the minimum and maximum values returned from the largest ROI. Thus, the mean, minimum, maximum, and standard deviation from the largest ROI were used for the subsequent analyses. The elasticity of the healthy parenchyma (on conventional US) was also measured. All the images were stored digitally.

Conventional US parameters and demographic data were used to build a predictive model discriminating benign from malignant lesions. Predictive capability of particular SWE parameters and their combinations were analyzed. Finally, a model based on both conventional US and SWE predictors was created. The first model (using only conventional US predictors) was built stepwise. The strength of all individual predictors was evaluated by means of univariate analysis (chi-square test or Fisher's exact factorial test in contingency tables). Then a multivariate logistic regression model was built. Its sensitivity and specificity were computed for different cut-off levels and the receiver operating characteristic (ROC) curve was plotted. All the tests were performed in STATISTICA, version 10.0, Statsoft Inc., Tulsa, CA, and MatLab R2013b, The MathWorks Inc., Natick, MA. The level of significance was always set to 0.05.

## 3. Results

### 3.1. Cohort Characteristics

Total of 96 benign and 28 malignant parotid lesions were included in the study; the distribution of diagnoses is summarized in Table 1.

Benign lesions other than pleomorphic adenoma and Warthin tumor included oncocytic adenomas, lipomas, lipomatosis, basal cell adenoma, nonsebaceous lymphadenoma, branchiogenic cyst, and chronic inflammation. In 6 patients with squamous cell carcinomas, the parotid lesions represented metastases from the other head and neck primaries. In remaining 2 patients, the primary was not identified. We considered that these squamous cell carcinomas originated in the parotid.

### 3.2. Conventional Ultrasound Parameters

A benign/malignant classifier was built using only conventional US parameters.  Table 2 summarizes the results and the statistical significance of the relevant parameters. Acoustic shadow was not used as it was observed in one patient only. Similarly, distal acoustic enhancement was observed in all but five patients. Therefore, this predictor was disregarded, too.

**Figure 2:**
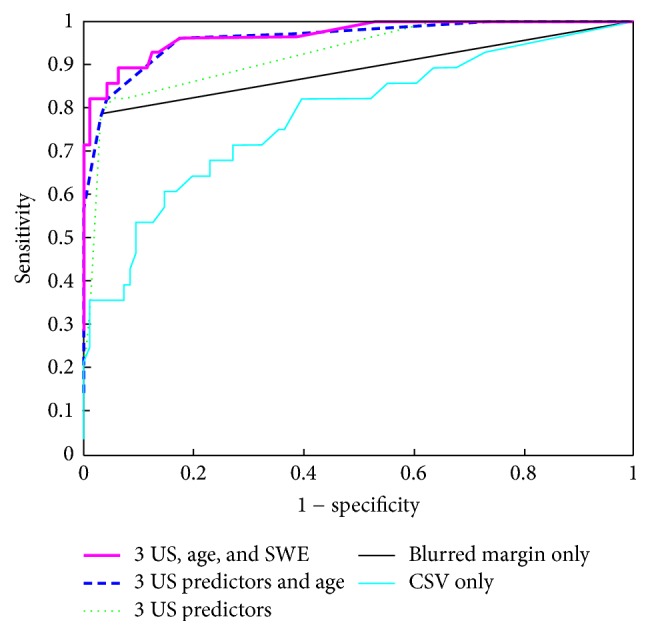
Five different models characterized by ROC curves were built to predict malignancy. All models were calculated as logistic regression classifiers. The bottom thin line corresponds to CSV as a single predictor (see the text for details). The thick line uses tumor delineation (blurred margin) as the only predictor. The dotted lines combine 3 US predictors, that is, tumor delineation, homogeneous echogenicity, and calcification presence. The dashed line combines these three US predictors with age ≥ 65, and the top thick line adds CSV parameter to these.

When building a predictor of malignancy, clear delineation of the lesion was found to have the greatest predictive power. It was possible to predict the malignancy of the finding by this predictor alone with as few as 6 false negatives (sensitivity of 22/28 = 79%) and 3 false positives (specificity of 93/96 = 97%). The addition of other 2 predictors (homogeneous echogenicity and calcification presence) to the model increased its performance only marginally (see the ROC characteristics of both these models in Figure 2). Adding enlarged cervical lymph nodes would not improve it at all.

### 3.3. Demographic Parameters

In our study, only the age was found to be a significant predictor of malignancy (*p* < 0.0001). The median age of patients with a benign finding was 58 years, whereas the median age of patients with malignant tumors was 68 years. Dichotomizing age at 65 years gives the best predictive power. Combining dichotomized age with the 3 US predictors (tumor delineation, homogeneous echogenicity, and calcification presence) described above yields improved ROC characteristics (see Figure 2, dashed line). Despite the superior ROC curve of the latter classifier, the optimal cut-off still produces 3 false positives and 6 false negatives in the presented study, which is the same performance as the model with blurred margin only.

### 3.4. Elastographic Parameters

Our SWE measurements show that malignant tumors tend to have higher maximal and lower minimal values. The minimum stiffness of many malignancies reaches 0.1 kPa, which is the lower technical limit of the US device. This limit value appeared in all anechoic regions which commonly represented cystic tumor components.

The maximum stiffness of the ROI alone is a reliable univariate predictor of malignancy (*p* = 0.0008). Surprisingly, the minimum stiffness is fairly good predictor as well (*p* = 0.01). Range and SD of the stiffness values should, therefore, be similarly indicative. However, with the minimum values approximating zero, the range would be very close to the maximum value. SD showed being a very good predictor (*p* = 0.0004). However, it can be affected by the size of ROI and it tends to suppress the overall minima and maxima in the data.

Thus, we newly created a coefficient of stiffness variability (CSV) as the ratio of the maximum and minimum stiffness values.

(1) $$\begin{array}{c} CSV\;\;\left(\;ROI\;\right)=\frac{\text{maximum of stiffness over the ROI}}{\text{minimum of stiffness over the ROI}}. \end{array}$$

The CSV is a strong predictor of malignancy (*p* < 0.0001); it discriminates malignant from benign findings better than any other SWE parameter. However, the ROC characteristics of the CSV predictor (Figure 2) are not even close to the ROC curves of the conventional US classifiers mentioned above.

### 3.5. Combination of Conventional Ultrasound and Elastographic Parameters

Our model combining three US parameters (lesion delineation, homogeneous echogenicity, and calcification presence), age ≥ 65, and the newly defined CSV elastographic predictor demonstrated 6 false negatives and 3 false positives (sensitivity of 22/28 = 79%, specificity of 93/96 = 97%) at the optimal cut-off value. With this cut-off, the predictive power of this model equals that one using blurred margin alone. However, it is possible to choose a higher cut-off value to produce only 4 false negatives (sensitivity of 24/28 = 86%) but 4 false positives (specificity of 92/96 = 96%).

**Figure 3:**
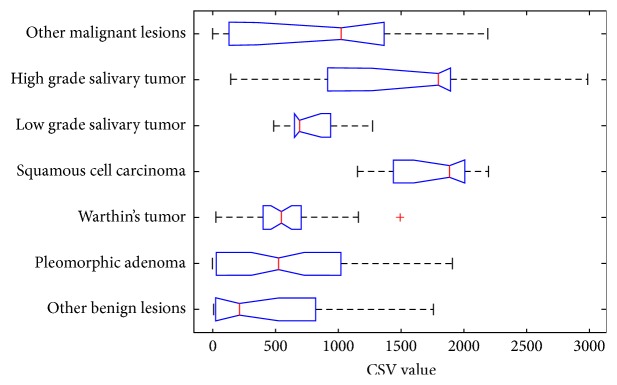
Breakdown of the CSV values according to the histological finding.

The lack of predictive power of SWE parameters can be explained by the breakdown of the CSV values according to the histological finding shown in Figure 3.

We observed high variance of CSV in the group of both benign and malignant lesions. Malignant findings generally exhibit higher CSV values but low grade salivary tumors are significantly less stiff than high grade tumors and squamous cell carcinomas. This explains the low malignant/benign predictive power of the CSV predictor. However, it also shows that SWE parameters (especially the CSV combination) can be used to make more specific predictions regarding the histology of the lesion.

## 4. Discussion

The main promise of elastography in prediction of biological character of a lesion is based on the premise that malignant tumors have higher stiffness than benign ones. It is assumed that the increased stiffness is caused by the tumor growth in a confined interstitial matrix resulting in the reactive interstitial fibrosis [14]. This works well in breast and in thyroid gland [11, 12]. However, the situation in parotid gland seems more complicated. This is caused by very variable histoarchitecture of salivary gland tumors which results in considerable variance in stiffness found both in our study (Figure 3) and previously published papers [4, 13]. Another complicating factor is the extremely wide range of elastographic values in pleomorphic adenomas (stiffness maxima may vary from 12.6 to 291.3 kPa) [6]. Due to its myxochondroid component, the stiffness of this benign lesion may be very high, overlapping that of malignant tumors.

Three studies used semiquantitative elastographic score (ES) [3, 4, 8] in the discrimination of parotid gland masses. Çelebi and Mahmutoglu found that with the exception of low grade carcinomas the ES did not improve the sensitivity and specificity of standard ultrasound in differentiation of benign from malignant lesions [4]. Bhatia et al. concluded that the elastography score had poor ability to discriminate the benign from malignant lesions, with pleomorphic adenoma causing major problems [3]. Wierzbicka et al. [8] found varying sensitivity and specificity depending on ES score. Similarly, we failed to demonstrate significant benefit of elastography in this oncology group (Figure 3).

Bhatia et al. enrolled just 5 malignancies and 55 benign lesions in his cohort. Therefore, the statistical comparison between the two groups was not possible [13]. Olgun et al. did not include any malignant tumor in their study at all [6]. In a group of 10 carcinomas, Wierzbicka et al. reported the sensitivity of conventional ultrasound in differentiation of benign from malignant lesions to be 93.8% and 62.5%, respectively. The authors studied quantitative SWE results (in kPa) corresponding to individual semiquantitative ES, which were previously the only outcomes of strain elastography. They found sensitivity of 80% and specificity of 45.5% for ES value of 2, 60% and 69.7% for the ES value of 3, and 40% and 97% for ES value of 4 [8].

The published studies using SWE in the parotid either did not engage in malignant lesions [6], had insufficient number of them [13], or evaluated the lesions by old ES [8]. This study is based on the largest patient cohort of all the studies dealing with parotid gland tumors evaluated by SWE so far [8, 13].

The results of the two studies using elastographic parameters along with the conventional and Doppler US were most similar to that of ours. Both authors combined various standard sonographic criteria to achieve the highest possible accuracy of the examination as we did. Klintworth and Badea [2, 7] assessed 57 and 20 parotid lesions, respectively, including 8 malignancies each. As one of the most accurate criteria they both assigned blurred margins, which is in concert with our results. Klintworth et al. described garland sign as significant for diagnosing malignant neoplasms [7]. Badea et al. found increased hypoechogenicity and increased stiffness and mobility “in block” in all malignant tumors. However these features occasionally appeared also in benign tumors [2]. Unlike our study, none of the authors used the quantitative SWE parameters.

We constructed a new elastographic parameter in our study, CSV. Similar principle is established in the SWE differential diagnosis of breast masses as mass-to-fat ratio [15]. Minima of stiffness instead of stiffness values of fat tissue are used in CSV. We regard this predictor as a better tool and recommend its use, rather than the semiquantitative ES score, in SWE measurements with results in kPa. It combines the maximal and minimal values to form a predictor, which is stronger in prediction of malignity, than both those values alone.

Tumor delineation proved to be the most reliable predictor of its dignity. However, we are aware of the fact that this predictor may have relatively high inter- and intraobserver variability.

Most malignant lesions showing benign US criteria in the classification by blurred (or clearly delineated) margin were categorized as low grade salivary tumors in our study. Their stiffness was relatively low, similar to that one of pleomorphic adenomas (Figure 3). Therefore, they were discernible from them neither by standard ultrasound criteria, nor by elastography. Fortunately, the recommended surgical therapy for pleomorphic adenoma and low grade salivary tumors is the same [16].

Our predictor combining three standard ultrasound parameters with age and SWE proved to be slightly better than the predictor based on blurred margin alone (Figure 2), but this may have been caused by overfitting. Taking into account the difficulties of combining the factors, almost no improvement of specificity (1 patient in our study, which means less than 1%) and just slight improvement of sensitivity, our recommendation is to evaluate parotid gland lesions by standard US criteria (mainly by blurred or clearly delineated margin) only.

## 5. Conclusion

Ultrasound in hands of an experienced physician may have fairly good specificity (97%) and sensitivity (79%) in preoperative diagnostics of parotid gland malignancies. Clear delineation of the tumor alone proved to be an excellent predictor. Shear wave elastography (coefficient of stiffness variability) is a significant predictor, too. However, adding this elastographic predictor to the conventional ultrasound ones improves the discriminatory power only marginally.

## Figures and Tables

**Table 1:** Summary of the diagnoses distribution.

	Count	Percent	Diagnosis
Benign	49	39.52	Pleomorphic adenoma
	33	26.61	Warthin tumor
	14	11.29	Other benign lesions

Malignant	8	6.45	Squamous cell carcinoma
	6	4.84	Low grade salivary tumor
	7	5.65	High grade salivary tumor
	3	2.42	Lymphoma
	2	1.6	Melanoma
	1	0.81	Sarcoma
	1	0.81	Neuroendocrine carcinoma

	*124*	*100*	*Total*

**Table 2:** Summary of the predictive power of individual conventional US parameters as a benign/malignant classifier. Chi-square test (or Fisher's exact test when more appropriate) *p* values provided for each predictor.

US parameter	Benign	Malignant	*p* value
Clearly delineated margin	93	6	**<0.001**
Blurred margin	3	22	

Not lobular shape	77	23	0.82
Lobular shape	19	5	

Heterogeneous echogenicity	39	19	**0.01**
Homogeneous echogenicity	57	9	

Mainly anechogenic	17	3	0.22
Mainly hypoechogenic	78	24	
Mainly isoechogenic	1	0	
Mainly hyperechogenic	0	1	

Absent calcifications	92	21	**<0.001**
Present calcifications	4	7	

Present cystic part	43	14	0.63
Absent cystic part	53	14	

Present septa in cystic part	11	1	0.10
Absent septa in cystic part	29	14	

Unilateral condition	85	28	0.06
Bilateral condition	11	0	

No vascularisation	32	5	**0.01**
1-2 vessels	23	9	
Peripheral vascularisation	23	1	
3 or more vessels	18	12	

Cervical lymph nodes not enlarged	88	18	**<0.001**
Cervical lymph nodes enlarged	8	10	
